# Image-Based Single Cell Sorting Automation in Droplet Microfluidics

**DOI:** 10.1038/s41598-020-65483-2

**Published:** 2020-05-26

**Authors:** Muhsincan Sesen, Graeme Whyte

**Affiliations:** 1 Heriot-Watt University, Institute of Biological Chemistry, Biophysics and Bioengineering, Edinburgh, EH14 4AS United Kingdom; 2 Present Address: Imperial College London, Department of Bioengineering, London, SW7 2AZ United Kingdom

**Keywords:** Optical imaging, Lab-on-a-chip, Image processing, Machine learning

## Abstract

The recent boom in single-cell omics has brought researchers one step closer to understanding the biological mechanisms associated with cell heterogeneity. Rare cells that have historically been obscured by bulk measurement techniques are being studied by single cell analysis and providing valuable insight into cell function. To support this progress, novel upstream capabilities are required for single cell preparation for analysis. Presented here is a droplet microfluidic, image-based single-cell sorting technique that is flexible and programmable. The automated system performs real-time dual-camera imaging (brightfield & fluorescent), processing, decision making and sorting verification. To demonstrate capabilities, the system was used to overcome the Poisson loading problem by sorting for droplets containing a single red blood cell with 85% purity. Furthermore, fluorescent imaging and machine learning was used to load single K562 cells amongst clusters based on their instantaneous size and circularity. The presented system aspires to replace manual cell handling techniques by translating expert knowledge into cell sorting automation via machine learning algorithms. This powerful technique finds application in the enrichment of single cells based on their micrographs for further downstream processing and analysis.

## Introduction

Cell populations are remarkably heterogeneous. Even considering a similarly tasked sub-population of cells; there exists significant variability in their morphological properties and more so in their active genes and regulations^1,2^. The recent genome-scale downstream capabilities (DNA^3^, RNA^4,5^) at the single-cell resolution provide unprecedented insight into cellular heterogeneity, especially in cases where rare cell populations are masked by bulk measurements. This calls for alternative upstream techniques of selecting single cells for analysis. Compared to conventional manual cell handling techniques, microfluidics offers significant reagent volume reduction and ease of automation with enhanced repeatability and detection accuracy on low-cost, disposable chips^6^. In this work, a droplet microfluidic system is developed to isolate single cells by image-based sorting. The platform aims to provide researchers with the methods to capture single cells of interest and study some of the deeper mechanisms involved in cell regulation and function.

There already exists well-established methods for the detection and isolation of single cells for analysis. Some of these techniques are label-free^7–12^ while others rely on fluorescent^13–16^ or magnetic labelling. Labelling might not be possible for some systems due to lack of biomarkers or potential cytotoxicity of the labels (e.g. DNA intercalators)^17^. Additionally, labels might be undesired if they interfere with the study such as in stem cell differentiation^18^. Well known techniques for single cell preparation are Fluorescence Activated Cell Sorting^19,20^, Magnetic Assisted Cell Sorting^21,22^, Fluorescence Activated Droplet Sorting^23^, Laser Capture Microdissection^24^ and Micromanipulation^14,25,26^. While some of these techniques can achieve high throughput, they are usually associated with high cost equipment and are less accessible, thus alternative methods are required^27^. Manual pipetting^1,28^ is usually the preferred method as it only requires an expert, a microscope and a pipette; however, manual pipetting is arduous and a waste of valuable resources. This work addresses the gap between high cost/throughput single cell preparation techniques and the manual pipetting alternative by automating single cell encapsulation in microfluidic droplets using image-based analysis. Other microfluidic approaches involving (single) cell detection and/or sorting for enrichment have been presented before; they are outlined in Table 1 to highlight the significance of this work. Major advantages of the presented system are (*i*) cells are encapsulated in droplets for individual screening, (*ii*) dual-colour imaging in brightfield and fluorescence, (*iii*) option to operate label-free with only brightfield imaging, (*iv*) capable of sorting cells based on micrographs either in brightfield or fluorescent modes, (*v*) easy microfabrication with single layer Polydimethylsiloxane (PDMS) and no metal deposition, (*vi*) real-time droplet volume measurement, (*vii*) real-time sorting verification, and (*viii*) machine learning ready. Example studies in which the presented technique could have been used are: Cells with intact nuclei (in brightfield) were selected in a forensic study for DNA analysis^25^; Circulating Tumour Cells (CTCs) and non-CTCs were selected (in fluorescence) based on markers as well as morphological criteria, cells with a small round shape having a nucleus without DNA fragmentations were preferred^29^.

**Table 1:** Significance of this work compared to relevant studies involving cell detection and single-cell preparation for analysis.

Detection	Label-free	Speed	Droplets	Sorting	Notes	ref.
Optical Scattering	N	30 Hz	Y	Solenoid	Sorting based on # of cells in a droplet	^96^
Electrical Impedance	Y	100 Hz	Y	—	Uses low conductivity buffer for droplets, can detect single cells.	^7^
Brightfield Imaging	Y	100 Hz	Y	DEP	Can detect droplets colonised by bacteria.	^62^
Optical Scattering	Y	—	Y	—	Can detect down to single bacteria.	^10^
Optical Scattering	Y	240 Hz	Y	DEP	Can detect droplets colonised by bacteria.	^31^
Optical Scattering	Y	160 Hz	Y	DEP	Works with chlorophyll-containing cells owing to autofluorescence.	^11^
Brightfield Imaging	Y	10 Hz	Y	DEP	Uses template-matching algorithm to identify circular or elliptic cells.	^57^
Brightfield Imaging	Y	250 Hz	Y	—	Automated inlet controls droplet volume and average # of cells.	^89^
Brightfield Imaging	Y	0.2 Hz	N	Solenoid	Sorts based on cell morphology.	^61^
Multi-Colour Imaging	N	100 Hz	N	Piezoelectric	Machine learning ready, highly capable system, sorts based on cell morphology.	^58^
Dual-Colour Imaging	Y&N	4 Hz	Yes	DEP	Machine learning ready, real-time sorting verification, sorts based on cell morphology.	This work

DEP: Dielectrophoresis, Y: Yes, N: No, Droplets: Does the study involve droplets?, Label-free: Is the detection label-free?

Droplet microfluidics, the study of two-phase flow in microchannels, has recently attracted considerable attention in single cell-omics owing to efficient co-encapsulation of single cells and barcodes for single-cell analysis^30^. Cyclic dispersion of a liquid in an immiscible carrier medium creates an emulsion rich with microreactors. These microreactors can be individually processed; for example, they can be loaded with cells^31^, picoinjected with other chemicals/targets^32^ and thermo-cycled for PCR^33^. Droplet processing requires precise control technologies^34^. Some examples are; droplet merging^35,36^, sorting^23,37^ and splitting^38,39^. These are usually achieved by using electrical^23,35^, acoustic^36–39^ or optical^31,40^ forces. In this study, dielectrophoresis (DEP) is used to shift aqueous droplets from a waste streamline to a keep streamline once a sorting decision is made. The reader is referred to the following papers^23,41–43^ for further reading on DEP and its safety on biological samples^44^.

In droplet microfluidics, cells can be individually encapsulated in nano to femtoliter microdroplets^45–47^. In a conventional droplet microfluidic single-cell encapsulation system, the rate at which doublets, triplets and beyond occur is dictated by Poisson statistics. This is a prominent roadblock in droplet-based single-cell sequencing technologies^48,49^. Two or more cells encapsulated in one droplet generate spurious transcriptome data, therefore disturb the results and invalidate the biological conclusions^50^. The occurrence of plural encapsulation could be decreased by lowering input cell concentration which reduces the effective rate of single cell encapsulation. Strategies to ensure single-cell encapsulation can be categorised as passive^51–56^ and active methods^23,57–59^. Collins *et al*.^60^ reviewed this subject in detail. One approach is to decrease the volume difference between droplets and cells. Abate *et al*.^53^ encapsulated 30 *μ*m diameter gel particles into comparable size droplets to improve single-particle encapsulation. Alternatively, droplets can be reduced to cell size using the jetting regime and sorted using size-based methods^51,56^. These techniques limit the amount of media or reagents that can be encapsulated in the droplet for metabolic activity and further studies.

In this work, an image-based microdroplet sorting platform is developed. This platform automatically images every droplet; images go through processing to extract valuable information pertaining to the contents of the droplet as well as the droplet itself. For example; (*i*) droplet volume, (*ii*) the instantaneous, 2D morphology and number of cells in the current droplet, (*iii*) the mean and variance of light intensity (brightfield or fluorescent) of the cells. This information is used to program the system to sort for droplets containing cells of interest. The system is flexible, therefore, holds potential for a wide range of applications such as isolating rare cell populations^50^, deterministic single cell encapsulation^60^, droplet size based sorting^54^, sorting yeast^61^ or bacteria^31,62^ cells, and encapsulating two cells to study cell-pair interactions^63^. To demonstrate the potential of the system, this paper details three example applications: (*i*) deterministic single cell encapsulation (red blood cells) by label-free sorting of droplets containing only one cell, (*ii*) label-free sorting of droplets containing single cells (red blood cells) based on cell micrographs, and (*iii*) machine learning assisted identification of fluorescently labelled K562 cells as single cells and clusters.

## System Overview

### Experimental setup

**Figure 1:**
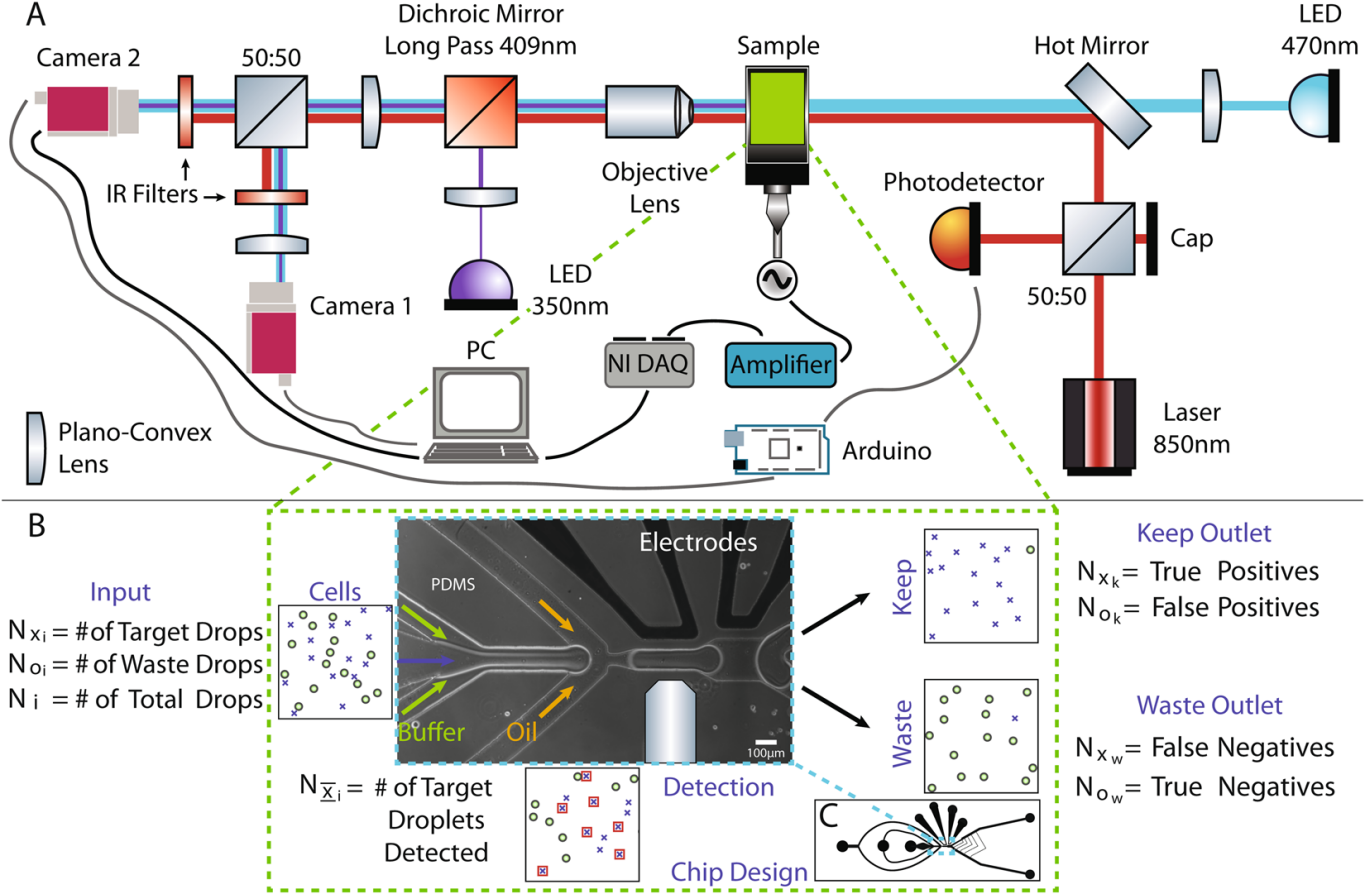
(**A**) Custom dual-imaging microscopy system schematic showing laser droplet detection components, and peripheral automation equipment. (**B**) Illustrative populations at the input (Cells), Detection zone, Keep and Waste outlets to define cell sorting performance parameters. Phase-contrast image of the microfluidic chip (channel borders are white) showing three inlets, the Y-junction and the electrodes for dielectrophoretic sorting. The width of the channel where detection takes place is 83 *μ*m. (**C**) Complete chip design is shown with three inlets to the left and two outlets to the right. The 4 ports at the top are for the electrodes.

A schematic overview of the system is shown in Fig. 1A. A custom built, modular microscopic imaging setup is employed in this study for sorting droplets containing the target samples, cells in this case. The sample is illuminated with a 470 nm LED (M470L3, Thorlabs, UK) and imaged using 10× and 40× objectives onto a camera (Mako U-130B, Allied Vision Technologies, Germany) for image processing. The fluids are infused into the device using syringe pumps (Legato 130, KD Scientific, USA). Image processing and control of peripheral equipment are performed using bespoke LabVIEW software. Droplet sorting is carried out using high-voltage fields generated by an amplifier (HVA Series, Ultravolt, USA) which receives the control signal from a data acquisition system (NI-DAQ, USB-6211, National Instruments). Image capture from the first camera is synchronised using a laser-scatter based detection system detailed below. An additional camera is used to visualise the sorting region of the device at a slightly lower resolution for validation and quality control, which is of paramount importance for cell sorting. Imaging from these two cameras is referred to as dual-imaging in the manuscript.

Dual-imaging is achieved by a 50:50 beamsplitter mounted on a kinematic stage (B4C, Thorlabs, UK) along with two cameras (Fig. 1A). This allows for real time image acquisition and verification for the sorting channels. By using a demagnifying lens (Fig. 1A) and adjusting the kinematic stage, the centre of the reflected image can be moved around the field of view and in this case, positioned to visualise the outlets to record images of which droplets are sorted into which outlets. There are three regions of interest (ROI) (see *Materials & Methods*) defined for the real time imaging camera 1 (Fig. 1A); one for counting droplets before they arrive the sorting Y-junction and one each for imaging droplets in the sorted and waste channels for verification and counting.

Camera 2 is used for capturing high-resolution images of droplets at a specific location before the sorting electrodes for processing and decision making. In order to ensure the droplet is repeatedly positioned at the optimum location in the device, a laser based backscatter droplet detection system is used; an 850 nm laser diode is positioned at the desired location within the device coaxial with the transmission light. The backscattered light is directed to a silicon photodiode detector (DET36A, Thorlabs, UK) via a 50:50 non-polarising beamsplitter cube (Fig. 1A). The output signal from the detector is fed into an analog input of an Arduino (Mega 2560, Arduino) via a voltage divider circuit. The custom programmed Arduino looks for a change in the signal caused by change in backscatter from the droplet-oil interface owing to refractive index mismatch. When a droplet is detected, the Arduino outputs a trigger signal to camera 2 to capture an image of the droplet. The laser light is blocked from being imaged on the cameras using two IR blocking filters (FES0800, Thorlabs, UK). With this technique, the location of the droplet is well defined which is important for image analysis as well as the timing of the electric field to sort droplets. The full width at half maximum of the laser spot is measured as 66 *μm* (see *Material & Methods*), slightly smaller than droplets with an average width of 80 *μm*.

To allow target droplets to be isolated from the others, two outlets from the device have been implemented. The *target* outlet has a smaller cross section and thus a higher fluidic resistance, resulting in droplets normally flowing down the waste channel of the device. When a keep decision is made, LabVIEW instructs the NI-DAQ to generate 20 square wave pulses (0–3 V, 500 Hz, 40 ms total duration) which are passed to the amplifier which amplifies it to 1.32 kV and applies to the electrodes within the device. This creates a high voltage AC field in the channel and a dielectrophoretic effect that pulls the target droplet to the high electric field intensity zone steering it to the keep channel, known as dielectrophoresis (DEP)^42^. The electric field is applied only for a short time (40 ms) resulting in the following droplets travelling into the waste channel and ensuring only one droplet is selected per pulse.

### Sorting performance metrics

Sorting performance measurements carried out in this study are mostly based on the discussions in the book by Lee *et al*.^64^. In order to understand these metrics, a few important parameters are defined. ${N}_{{x}_{i}}$ and ${N}_{{o}_{i}}$ are the numbers of target and waste droplets at the input, respectively (Fig. 1B). They add up to the total number of droplets input into the device, *N*_*i*_.

${N}_{{\overline{\underline{x}}}_{i}}$ is the number of target droplets detected by the imaging algorithm. ${N}_{{x}_{k}}$ and ${N}_{{x}_{w}}$ are the target droplets while ${N}_{{o}_{k}}$ and ${N}_{{o}_{w}}$ are the waste droplets collected in the keep and waste outlets, respectively (Fig. 1B). They are also known as true positives, false negatives, false positives and true negatives in order of appearance. Finally, *N*_*k*_ and *N*_*w*_ are the total number of droplets in the keep and waste outlets. Efficiency is defined as^64^:

1 $${\rm{Efficiency}}=\frac{{N}_{{x}_{k}}}{{N}_{{\overline{\underline{x}}}_{i}}}$$

Efficiency measures the efficiency of the sorting system, it is defined as the ratio of the number of droplets containing single cells in the keep outlet to the number of droplets detected as containing single cells. Occasionally, the electrical field timing or intensity prevents a successful steer.

2 $${\rm{Purity}}=\frac{{N}_{{x}_{k}}}{{N}_{k}}$$

Purity is the ratio of droplets containing single cells in the keep outlet to the total number of droplets steered into the keep outlet^64^.

3 $${\rm{Yield}}=\frac{{N}_{{x}_{k}}}{{N}_{{x}_{i}}}$$

Yield indicates how many targets from the inlet are captured in the keep outlet^64^. In the context of this study, it is the number of droplets containing single cells captured in the keep outlet divided by the statistical estimation of the number of droplets that should contain single cells.

4 $${\rm{Enrichment}}=\frac{{N}_{{x}_{k}}}{{N}_{{o}_{k}}}/\frac{{N}_{{x}_{i}}}{{N}_{{o}_{i}}}$$

Finally, enrichment quantifies how much the original sample was enriched with target cells; it is the ratio of target to waste droplets at the keep outlet divided by the same ratio at the inlet^65,66^.

### Image analysis and decision making

**Figure 2:**
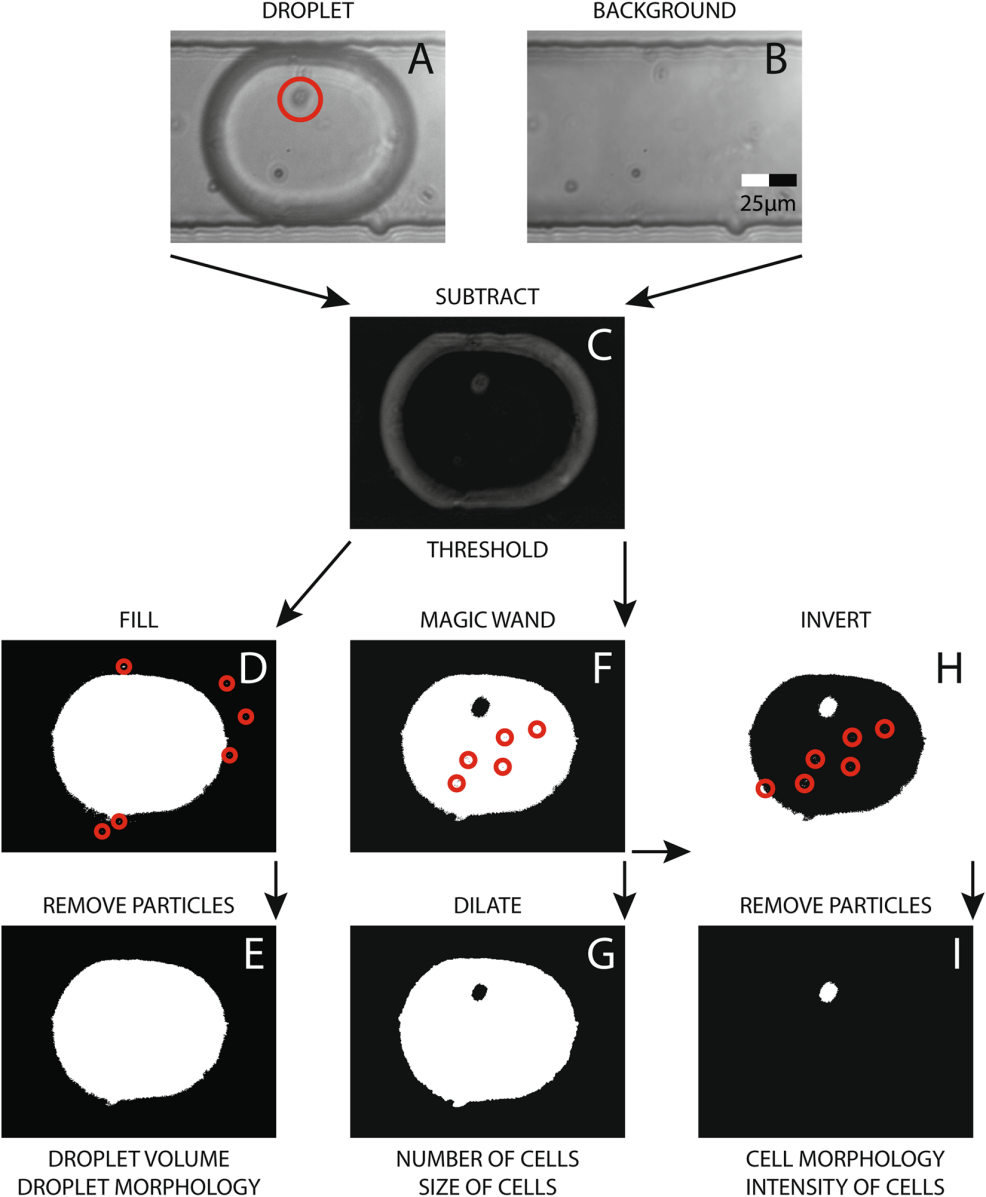
Image analysis and manipulation flowchart starting with background subtraction, thresholding and denoising to obtain cell and droplet parameters such as morphology and count.

When a droplet image (Fig. 2A) is captured, it undergoes several steps of image manipulation for cell identification and extraction of other relevant parameters. Firstly, the on-demand recorded background image (Fig. 2B) is subtracted from the captured image to remove static features such as the channel walls and debris (Fig. 2C). Next, an intensity threshold (10 for 8 bit images) is applied to the image to isolate the entire droplet in the foreground; this manipulation generates salt and pepper noise as indicated by red circles in Fig. 2D. These are then removed with ‘IMAQ Remove Particle VI’^67^ to finally the get the outline of the droplet (Fig. 2E). From this binary image mask, numerous parameters are extracted^68^; important among those are droplet area, circularity, length and width.

In parallel, the subtracted image goes through a higher threshold (12 for 8 bit images) that only highlights the edges of the droplet. Then, ‘IMAQ MagicWand VI’^69^ (similar to Microsoft Paint’s ‘Fill with colour’ tool) is used to fill the inside of the droplet excluding any cells that may be in the droplet. This also leads to some salt and pepper noise shown by the red circles Fig. 2F which is removed by dilating the image twice (Fig. 2G) using ‘IMAQ Morphology VI’^70^. This binary image mask is used to obtain the number of cells in the droplet as well as the size of the cells using ‘Area of Hole’ parameter^68^, which counts the number of pixels in the dark region inside the droplet. This value can later be converted to *μm*^2^ using pixels-to-microns ratio of the imaging system. It should be noted that the size of non-circular cells will significantly vary depending on the instantaneous orientation of the cell during image capture.

Finally, ‘IMAQ MagicWand VI’^69^ output (Fig. 2F) is inverted (Fig. 2H) and both the border and particles are removed to obtain cell binary mask as shown in Fig. 2I. ‘IMAQ Particle Analysis VI’^71^ automatically calculates various visual parameters pertaining to the cell(s) using this binary mask (Fig. 2I). A list of these parameters along with graphical examples can be found on their website^68^. In this study, some of these parameters were used to demonstrate how cells of interest could be selected using these readily available tools. Importantly amongst these, this study uses ‘Number of Particles’ to determine the number of cells in a droplet, ‘Area’ to calculate droplet volume, ‘Ellipse Ratio’ & ‘Waddel Disk Diameter’ for selecting circular single cells and rejecting erroneous masking. Furthermore, ‘Heywood Circularity Factor’ & ‘Area’ were used as input for supervised machine learning to sort between single cells and clusters. This binary image (Fig. 2I) could also be used to extract cell intensity characteristics from the original image (Fig. 2A). For non-spherical cells that are constantly rotating inside the droplet, ‘Max Feret Diameter’ (the longest line that could be drawn on the particle)^68^ could be used to find the biggest diameter which should provide an insight into size of the cell population. All the above-mentioned, image-based properties of cells or droplets can be used individually or in combination with each other to enrich cell populations of interest which make this system versatile and powerful. For example, inherent droplet volume variability during droplet production has been shown to produce misleading results especially in droplet digital polymerase chain reaction (ddPCR)^72^. With the presented system, droplets could be sorted for their size to improve the accuracy of ddPCR measurements.

The decision making is performed real time with access to all the parameters explained above. Two main strategies are used in the decision making; logic gating and supervised machine learning. Logic gating is used for simpler scenarios such as single-cell encapsulation in droplets - the logic gate is programmed to keep all droplets where the number of detected cells is equal to one. This can be extended to include a certain lower and upper limit for apparent cell or droplet size, a cell circularity threshold and as many more as required. In supervised machine learning, all or some of the available parameters are fed as input to the classifier along with their keep or waste labels determined by experts in their field. Once the classifier is trained with the initial set of data, it can sort new samples that aren’t suitable for logic gating or with better purity compared to logic gating.

### Throughput

Throughput was a function of the inlet flow rates in this study. During experiments, LabVIEW recorded the total number of droplets detected by the system and the total duration of the experiment. The ratio of these values was used to determine the throughput for each experiment. At 3.5 Hz, where experiments were conducted, sorting efficiency of 100% was achieved while at 4.5 Hz, sorting efficiency dropped to 80%, therefore we report a throughput of 4 Hz as shown in Table 1.

Real-time image-based sorting is inherently a low throughput technique as it involves the acquisition and processing of image data on the order of megapixels. Since the images are acquired during flow; the most important parameters for a blur-free image are exposure time (*t*_*e*_), pixel size (*s*_*p*_) and flow velocity. If the target is not allowed to traverse more than one pixel during the exposure time; the maximum velocity can be found by *V*_*max*_ = *s*_*p*_/*t*_*e*_. This is the first rate-limiting step in this setup. This can be improved by increasing the pixel size - sacrificing resolution - or decreasing the exposure time - sacrificing light - both of which are equally precious.

In this work, the pixel size was measured by acquiring a still image of a calibration target (R1L3S2P, Thorlabs, UK) and measuring the number of pixels between 10 divisions (100 *μ*m). Pixel size for the brightfield camera (Mako U-130B, Allied Vision Technologies, Germany) was 250 nm and the pixel size for the fluorescent camera (340M-USB, Thorlabs, UK) was 390 nm. *V*_*max*_ was calculated from *V*_*max*_ = *s*_*p*_/*t*_*e*_ as 1 *mm*/*s* and 0.8 *mm*/*s*, for the cameras, respectively. During optimisation experiments, the flow rate was increased until the image quality and sorting efficiency was acceptable. At 75 *μ*L/hr of oil and 2 *μ*L/hr of aqueous flow, the throughput was measured by the LabVIEW algorithm as 3.5 Hz. The corresponding flow velocity was calculated as 8 mm/s using the cross-sectional area at the detection region.

The second rate-limiting step in this study was the selected DEP actuation duration, 40 ms, limiting the throughput to 25 Hz. There was uncertainty in the droplet location when it was detected, also in the time between droplet detection and DEP triggering as well as in droplet velocity. 40 ms was chosen to minimise erroneous sorting events. Sciambi and Abate^73^ showed that droplets could be sorted at 30 kHz using DEP, therefore DEP sorting is not considered a future barrier in image-based droplet sorting.

The third rate-limiting step was the LabVIEW program. Timing experiments carried out with LabVIEW including the usage of supervised machine learning algorithms showed a 20 ms (50 Hz) acquisition-to-decision time on an average PC. This could be improved by using a field-programmable gate array (FPGA) to acquire images and to process them for sorting decision. The work by Nitta *et al*.^58^ focus on maximising throughput and reports a throughput of 100 Hz by using state-of-the-art equipment including an FPGA.

The fourth rate-limiting step was the cameras that were used in this study. For brightfield imaging, Mako U-130B (Allied Vision Technologies, Germany) with 168 fps maximum frame rate at full resolution was used. For fluorescent imaging, 340M-USB (Thorlabs, UK) with 200 fps maximum frame rate at full resolution was used. For completeness, next factors were the NI-DAQ (USB-6211, National Instruments), the Arduino (Mega 2560, Arduino), and the amplifier (HVA Series, Ultravolt, USA); all of which can achieve throughputs on the order of kHz and more.

## Results & Discussion

### Cell concentration

A crucial parameter for single-cell encapsulation in droplets is the concentration of cells, CC, in the aqueous phase. This variable directly affects the number of empty droplets, droplets containing single cells, two cells and so on. *λ*, the mean number of cells in a droplet across many, can be calculated by multiplying cell concentration and mean droplet volume, *V*:

5 $$\lambda =CC[cells/mL]V[mL]$$

**Figure 3:**
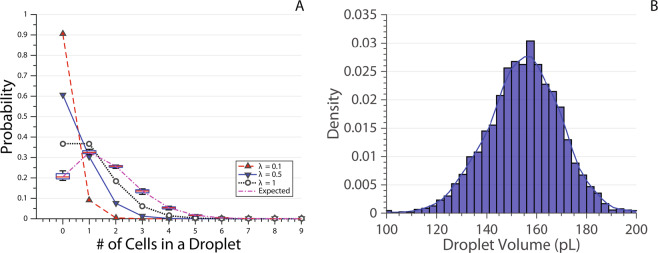
(**A**) Probability of the number of cells in a droplet calculated for *λ* = 0.1 (red up triangle), 0.5 (blue down triangle) and 1 (white circle). Box plot of expected probability values calculated for each experiment using experiment specific red blood cell concentration (CC) and mean droplet volume (V) in Eq. 5. CC is calculated before the experiment by a haemocytometer, V is calculated after the experiment by averaging the volume of all droplets. The box plot is denoted ‘Expected’ values because the real CC value would be less due to cell sedimentation. The lines are joining data points for visual purposes. MATLAB’s default box plot is used, the red line shows the median, and the box denotes the 25^*th*^ and 75^*th*^ percentiles. The error bars (whiskers) extend to cover all data points. (**B**) Droplet volume recorded across different experiments carried out on multiple chips.

Doublets, triplets and beyond are highly undesirable false positives for single-cell studies, therefore techniques like inDrop^74^ and Drop-seq^75^ reduce the cell concentration to achieve a λ between *λ* = 0.1 and *λ* = 0.5 where most droplets are empty, between 10% and 30% have single cells and less than 10% has doublets (Fig. 3A).

With the system presented here, the inlet cell concentration was adjusted to render *λ* ≈ 1 during red blood cell (RBC) sorting. As shown in Eq. 5, droplet volume is required for calculating *λ*; area of the droplet, recorded throughout the experiments using the binary image in Fig. 2E, is multiplied with the height of the microfluidic channel to obtain droplet volume (cylindrical assumption). Normalised (Probability Density Function) droplet volume from the reported experiments with RBCs are shown in Fig. 3B. The mean droplet volume across all experiments was calculated as 155pL.

The image analysis algorithm can identify single target cells encapsulated in a droplet with precision. In this way, it presents an opportunity to maximise the probability of single-cell encapsulation, *P*(1). Empty droplets, *P*(0), doublets, *P*(2), triplets, *P*(3), and even more cells in single droplets are biased to the waste outlet. The probability of encapsulating *k* cells in the next droplet, *P*(*k*), is given by^76^:

6 $$P(k)\approx {e}^{-\lambda }\frac{{\lambda }^{k}}{k!}$$

The peak for *P*(1) occurs when *λ* = 1 where 36.8% of droplets are expected to contain single cells (Fig. 3A). Cell concentration (CC) was calculated using a haemocytometer before loading the input cell solution to a syringe. During experiments, droplet volumes were detected by the custom LabVIEW software and averaged to obtain mean droplet volume (V). CC and V were used in Eq. 5 to calculate the expected probability values for four independent experiments (also see subsection ‘Data Map’ in ‘Materials & Methods’). The results are denoted ‘Expected’ in the legend (‘Expected’ because the final CC is lower due to cell sedimentation) and shown as box plots in Fig. 3A. *λ* turned out to be more than planned as this is an iterative process which involves pipetting of microliters of whole blood into the buffer solution. It is important to note that working with *λ* < 1 is more favourable than working with *λ* > 1 as this just increases the chance of doublets versus empty droplets.

### Sorting performance results

To validate the system is capable of sorting droplets containing a single cell with high accuracy, four 1-hour sorting experiments were carried out on different days with disposable microfluidic chips. In each of these experiments, human blood was diluted to the desired cell concentration and infused as the aqueous input for the sorting microfluidic chip (see *Materials & Methods*).

**Figure 4:**
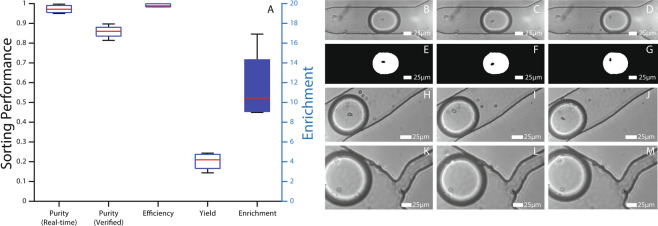
(**A**) Sorting performance parameters during single red blood cell encapsulation are calculated for each experiment separately and presented as box plots. MATLAB’s default box plot is used, the red line shows the median, and the box denotes 25^*th*^ and 75^*th*^ percentiles. The error bars (whiskers) extend to cover all data points. Purity is both calculated real-time during the experiment according to Eq. 2 and human verified after the experiments. A doublet that is detected as a single cell by the imaging analysis is an example of the discrepancy between the two. Efficiency, Yield and Enrichment are calculated as stated in Eqs. 1, 3 and 4. Enrichment box is coloured blue and its values are shown on the right Y-axis. (**B**–**D**) Example micrographs captured for cell detection. (**E**–**F**) Binary image masks (see Fig. 2G) calculated by the image manipulation algorithm. (**H**–**J**) Example micrographs of the sorted droplets into the keep channel. (**K**–**M**) Example micrographs of the droplets going into the waste outlet.

As discussed above, the use of dual-imaging setup allows real-time verification of the sorting by tracking where each droplet leaves the sorting region. This capability was used to evaluate the droplet sorting ability of the system. Whenever a target droplet is detected, and the pulse is applied, the timestamp is recorded. If a droplet is then detected in the keep outlet within 200 ms, the system records this event as a successful sorting event. Efficiency is the parameter used for reporting the incidents where there were timing issues, or the electrical signal failed to induce a strong DEP (see Eq. 1). As can be observed from the Efficiency results in Fig. 4, this does not happen very often (i.e. ~100%) indicating that the sorting system is working up to its design standards. Out of 3126 actuation events, 25 droplets were missorted during experiments due to DEP handling errors.

Whenever a droplet ends up in the keep channel without being identified as containing a single cell (i.e. without electrical signal), this is recorded as a false positive, ${N}_{{o}_{k}}$. Purity (Real-time) (Fig. 4) measures the ratio of sorting events to the total number of droplets in the keep channel (see Eq. 2) and is >95%. To quantify cell identification errors and find the real number of true positives, all droplet images were looked at by humans and tagged as containing single or multiple cells. The results of this are shown as Purity (Verified) in Fig. 4 which is ≈85%. This value also includes the false positives recorded by the software (i.e. Purity (Real-time) ≥ Purity (Verified)).

It was observed that the majority of cell identification errors were caused by the boundary effect. For example, when a second cell is located at the very boundary of the droplet, it can become merged with the droplet boundary during image processing. The differing refractive indices of the oil and aqueous solution lead to refraction at the boundary which gives the droplet its dark edge as observed in Fig. 2A. This dark edge can mask objects at the boundary. Although this is important for detecting the droplet itself, it makes it difficult for the program to see the cells that are on the edge of the droplet at the imaging moment. By adjusting the focus of the lens, the dark ring could be made bigger or smaller which gives the user the option of optimising towards Purity or Yield.

Yield is the parameter for measuring the system’s performance in identifying all the targets (see Eq. 3). The calculation for the number of target droplets at the input, ${N}_{{x}_{i}}$, is carried out by multiplying P(1) with the total number of droplets in the experiment to find how many of them are expected to contain single cells. That is then compared to how many droplets are identified as having single cells in them. Yield of the system is around 20% which is unexpectedly low (Fig. 4). This is believed to be due to cell sedimentation in the syringe which decreases the cell concentration entering the device and consequently P(1). On top of that, some droplets with single cells in them are not detected by the system (lensing effect) contributing to this decrease in Yield. In another set of experiments, Yield was increased to 40% by increasing flow rate (decreases cell sedimentation) but the system is not optimised at these flow rates so these results are not shown.

Finally, Enrichment is the parameter used to compare the initial state of the sample to the final state (see Eq. 4). Enrichment varies a lot more as it’s defined as a ratio of ratios. The reported system was shown to enrich samples by 10 times (Fig. 4) and more. Enrichment can be improved greatly as the throughput and therefore yield is increased. For example, Enrichment as high as 300 was observed when yield was doubled up with the higher flow rate (results not shown).

### Application - transmission imaging

**Figure 5:**
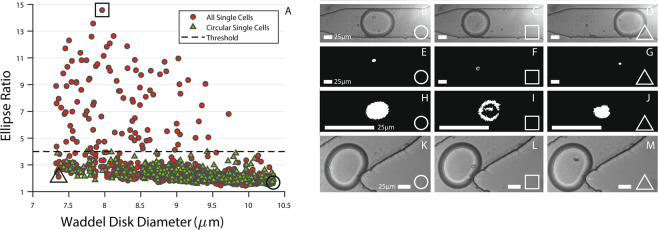
(**A**) The Waddel Disk Diameter and Ellipse Ratio of every single red blood cell encapsulated in a droplet that the system decided to sort for two experiments. In the’All Single Cells’ experiment, there were no constraints based on cell micrographs whereas in the’Circular Single Cells’ experiment, the ellipse ratio was capped at 4 with logic gating. (**B**–**D**) Micrographs for the circle, square and triangle data points as marked on (**A**). (**E**–**G**) Binary image masks (see Fig. 2I) calculated for the three selected data points. (**H**–**J**) Zoomed in versions of (**E**–**G**). The misidentification of the red blood cell in the square data point can be seen in (**I**). By logic gating, erroneous data points are excluded. (**K**–**M**) Micrographs of the droplets after being sorted into the keep outlet. All scale bars are 25 *μ*m.

Configuring the sorting software for a specific application is very easy. To demonstrate decision making based on cell micrographs, two experiments were performed. In the first experiment, the sorting automation software was configured to capture all single red blood cells (RBCs) regardless of their shape and size (Fig. 5A). In the second one, logic gating was used to reject RBCs that had an Ellipse Ratio higher than 4. Ellipse Ratio of a perfect circle is 1 and it increases as the object is more elongated. It’s calculated as the ratio of major to minor axis length. Due to the unique shape of RBCs (biconcave disc), their instantaneous orientation inside droplets vary from a rod-like ellipse to a circle. While it may seem pointless to sort RBCs from RBCs, we are using this variation to demonstrate the system’s capability of decision-making via morphological differences in cell micrographs.

The Waddel Disk Diameter^68^ (in *μm*) and Ellipse Ratio^68^ of single RBCs encapsulated in droplets that the automation software decided to keep during these experiments are shown in Fig. 5A along with the chosen threshold. The red circle data points indicate all RBCs and the green triangular data points show selected RBCs. As can be seen from the results, the system rejected cells where the ellipse ratio was larger than 4 in the hunt for non-erroneous, circular single cells. Three special data points marked with a circle, square and a triangle (Fig. 5A) were selected as examples and shown in Fig. 5B–M. Figure 5B–D show the original image captured in brightfield (see Fig. 2A). Figure 5E–G are after dilating (see Fig. 2G); Fig. 5H–J are zoomed in version of Fig. 5E–G and Fig. 5K–M show images of the same droplets after being sorted into the keep channel. By thresholding Ellipse Ratio to 4, the system was instructed to sort for circular single cells (Fig. 5A) and discard less circular ones which exhibit erroneous masking of the cells within droplets (Fig. 5I).

This application study demonstrates that the presented label and aerosol-free, transmission imaging based technique could detect visual differences in cell populations. This capability has been used, for example, in separating yeast cells based on their morphologies^61^ which is critical in understanding cell division cycles. Yeasts that are budding, given that their orientation is right, would have a much larger ellipse ratio compared to ones that aren’t. Setting up similar constraints in the presented setup would allow them to be sorted easily and efficiently. While the system is limited by instantaneous orientation of the imaged cells, this could be alleviated by aligning such non-circular cells using inertial forces^77^.

### Application - fluorescent imaging and machine learning

In another application study, slight modifications to the system (see *Materials & Methods*) allowed camera 1 to carry out fluorescent imaging while camera 2 continued imaging in transmission mode for sorting verification. In this mode, K562 cells stained with Hoechst, a fluorescent dye that binds to DNA, were imaged within droplets in real-time using the presented microfluidic system.

Existence of fluorescent nuclei against the dark background was easily detected with the high signal-to-noise ratio similar to *Droplet Detection with Region of Interest* discussed in *Materials & Methods* section. After detection of cells, an adaptive intensity threshold (see *Image Analysis and Decision Making*), *T*_*f*_, was applied to the fluorescent images according to *T*_*f*_ = 0.25*I*_*max*_ where *I*_*max*_ is the maximum intensity of the acquired image. The appearance and number of cell nuclei in a single droplet was accessed via this thresholding.

To prevent confusion, the wording for this section will be as follows: *labelling* is carried out by humans, *classification* is done by supervised machine learning (MATLAB) and *sorting* by image manipulation and boolean logic gating (i.e if there’s one cell in the droplet) in LabVIEW.

**Figure 6:**
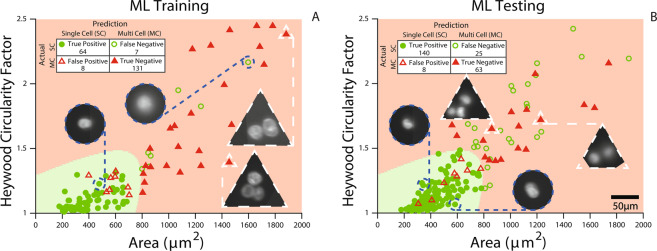
(**A**) Experimental results showing area and circularity features of fluorescent K562 cells imaged in droplets during flow. They are labelled as containing single (circle) or multiple cells (triangle). This data is used for Machine Learning Training (ML Training). The green and red areas are determined by supervised machine learning to select single K562 cells. (**B**) Experimental results from another experiment using the green and red areas from part (**A**) for sorting. The inset tables show the results of supervised machine learning predictions (ML Testing). The circle and triangle insets show example fluorescent images containing single and multiple cells in droplets. There are some true negatives that lie beyond the boundaries of the plotted graph and are not shown.

In the first experiment, the system sorted all the droplets it identified as containing a single cell. However, it wasn’t successful all the time; there were cell clusters amongst these selections. The importance of clusters in cancer metastasis has been recently highlighted^78^. In one study, a microfluidic chip for capturing circulating tumour cell clusters was reported^79^. In a recent study, clustering of MCF7 cells led to reduced detection efficiency^80^. In this study, to separate clusters from single cells logic gating was not enough, therefore machine learning (ML) principles were employed. Images from this first experiment were labelled as single cells (circle) or multiple cells (triangle) and used as ML Training data. The scatter plot for this experiment can be seen in Fig. 6A.

The informative, discriminating and independent features of this sample were selected as circularity and size (measured by area) as plotted in this figure. For circularity, Heywood Circularity Factor is used^68^. Heywood Circularity Factor of a perfect circle is 1 and it increases as the object is more irregular. Clusters were observed to be less circular (higher circularity factor) and larger in size. These features along with their respective labels were fed into MATLAB’s supervised machine learning (Classification Learner) tool. A quadratic SVM (Support Vector Machine) fit was chosen for segregating clusters from single cells. The area in green (close to origin) is the area identified by machine learning as the optimum region for isolating single cells (see Fig. 6A). Misclassified data points are shown as hollow whereas correct predictions are filled. The circle and triangle insets exemplify some of the single cells and clusters imaged within droplets during flow.

In the second experiment (Fig. 6B), the same green and red areas identified by MATLAB was used to segregate single cells (circles) from cell clusters (triangles). This demonstrates how the expert classification from the first experiment (Fig. 6A - ML Training) could be used to drive the decisions successfully in another experiment (Fig. 6B - ML Testing); the detailed numerical results are outlined in the inset tables. Most importantly, these two experiments show how expert identification of a specific cell subpopulation could be used to train our sorter for further experiments. To generalise and summarise the workflow:

1. The system is initially run in imaging cytometry mode (Fig. 6A).
2. The cells of interest are labelled by an expert (Fig. 6A).
3. The labels are used to train supervised machine learning classifiers (Fig. 6A).
4. The system is run with the classifier to enrich cells of interest for collection and further downstream processing (Fig. 6B).

## Conclusion

Presented here is a two-phase microfluidic system capable of real-time, dual-camera imaging (brightfield & fluorescent) within droplets during flow. The acquired image is processed to decide for sorting cells based on adjustable/programmable properties. The sorting is carried out by applying a short, high voltage pulse to the electrodes embedded within the chip that induce a well-established technique, dielectrophoresis. This system incorporates a laser-triggered droplet detector ensuring the timing for the electrical actuation as well as enhancing the throughput. The system is thoroughly characterised by quantifying the sorting performance.

To showcase its capabilities, the system is programmed to address the Poisson loading problem by sorting for droplets containing a single cell: a highly desired preparation technique for single-cell sequencing. In this way, initial cell concentration could be optimised for maximising the probability of single-cell encapsulation without worrying about plural encapsulation. The sorting performance of the system is monitored real-time with the aid of the second camera that observes the outlets and saves images of sorted droplets for verification purposes. Overall, it’s established that the system performs with high purity, efficiency and enrichment.

To further demonstrate powerful application with this technique, the system was slightly modified for high resolution fluorescent imaging of cells within droplets during flow. By performing an initial cytometry run, single cells of interest were identified and fed this information to supervised machine learning algorithms to optimise the selection. This selection was used in the second experiment to sort the cells of interest, single cells from clusters in this case. The presented system offers a reliable technique for partitioning cell sub-populations based on their morphologies and visage. These partitions could then be further characterised for understanding their biological functions^81^. The former is often performed by a trained expert who manually picks the cells of interest using a pipette.

Overall, the developed system performs well in sorting cell populations, efficiently producing highly pure and enriched samples at the outlets for further observations such as single-cell RNA sequencing. Moreover, the microfluidic chip contains a secondary aqueous inlet that could be used for introducing chemicals like cell lysis buffer for examining and imaging cells going through rapid changes, such as activated immune cells^82^. The presented system will not be able to compete with high-throughput sorting techniques such as fluorescent activated cell sorting as the full image takes longer to acquire and process, however, it can be used as a complementary technique when small cell populations need to be analysed or when labelling is undesired or when the image data presents a unique opportunity for sorting cell populations. The presented platform could be strengthened by additional sensing modules to aid in the decision making such as differential detection photothermal interferometry^83^, digital holographic microscopy^84^, whispering gallery mode resonators^85^, refractive index cytometry^86^ or impedance cytometry^87^.

## Materials & Methods

### Device fabrication

Microfluidic channels were patterned onto silicon wafers using SU-8 2025 (MicroChem Corp., USA) photolithography^88^. Channel height was measured via cross-section microscopy as ≈30 *μ*m. PDMS was cast onto the moulds, degassed, cured, peeled, ported and bonded onto glass slides or coverslips as described elsewhere^89^. The chips were heated to 150 C for indium gallium arsenide (InGaAs) solder wicking into the electrode channels via capillary action^90^.

### RBC preparation

Experimental protocols involving red blood cells (RBCs) were approved by the Heriot-Watt School of Engineering and Physics Sciences Ethics committee. Whole blood was obtained by finger prick from healthy volunteers in accordance with Heriot-Watt University ethical guidelines with informed consent. RBC samples were prepared by adding 3 *μ*L of whole blood into 2.65 mL of buffer solution consisting of 28.1% OptiPrep^TM^ in Phosphate Buffered Saline (PBS -Mg -Ca) with 1% Pluronic. This buffer was produced to have a density of 1.095 g/mL to match that of RBCs^91^ to prevent sedimentation as well as cell adhesion and clumping. The final sample was further diluted (1:10) and loaded into a haemocytometer for counting: ≈1.7 × 10^6^ cells/mL (diluted), ≈17 × 10^6^ cells/mL (input sample). Finally, samples were loaded into a syringe for performing experiments.

### K562 preparation

K562s (human erythromyeloblastoid leukemic cells) were cultured with standard culturing techniques^92^. Hoechst 33258 stain was added to K562 cells suspended in culture media in a test tube (1:100 (v:v)). The tube was kept in the dark for 10 minutes while cells were counted in a counting chamber (haemocytometer) (≈10^6^ cells/mL). The sample was then centrifuged at 6000 RPMs for 2 minutes and cell culture supernatant was removed before they were resuspended in a buffer solution consisting of 16% OptiPrep^TM^ in Phosphate Buffered Saline (PBS -Mg -Ca) with 1% Pluronic. The final suspension was diluted (1:10) for a final cell counting: ≈5.5 × 10^5^ cells/mL (diluted), 5.5 × 10^6^ cells/mL (input sample).

### Experimentation

All reported experiments are carried out for an hour without any human intervention such as rotating the syringe to prevent cell sedimentation. Polytetrafluoroethylene (PTFE) tubing was used to connect microfluidic inlet ports to 1 mL syringes loaded onto syringe pumps (Legato 130, KD Scientific, USA). An engineered fluid (3 M^TM^ Novec^TM^ 7500) stabilised by 2% surfactant (Pico-Surf^TM^ 1, Sphere Fluidics, UK) was used as the oil phase while the dispersed phase was either the cell-specific buffer solution or DI-water. Flow rates for the oil and aqueous inlets were 75 *μ*L/hr, 1 *μ*L/hr and 1 *μ*L/hr, respectively.

### Data map

In this section, the details of the data used in figures will be outlined. Every experiment has a unique ID associated with it. All experiments except 1140 and 1150 are carried out with the brightfield configuration using red blood cells. Experiments 1140 and 1150 are carried in the fluorescent configuration with K562 cells. Figure 3A ‘Expected’ *λ* values are calculated using MATLAB’s default box plot tool with 4 values. These 4 values are calculated using cell concentration (CC) and mean droplet volume (V) in Eq. 5. CC is counted with a haemocytometer before experiments 1085, 1090, 1127 and 1130. V is calculated by averaging the volume of every droplet detected by the LabVIEW software during experiments 1085, 1090, 1127 and 1130. A total of 41884 droplets were processed during these experiments. Figure 3B is a histogram plot of the volumes of 3279 droplets detected as containing single cells from experiments 1085, 1089, 1116, 1126 and 1130. Figure 4A is the box plot of sorting performance parameters from experiments 1085, 1090, 1127 and 1130. A total of 41884 droplets were screened, 3126 droplets were detected to contain single cells. Figure 5A, circle (red) data points are from experiment 1085 containing 823 single cell detections. Figure 5A, triangle (green) data points are from experiment 1089 containing 397 detections with the logic gating applied. Figure 6 data is from experiments 1140 and 1150, the details of which are shown in the inset table.

### Fluorescent imaging

For fluorescent imaging, the following changes were carried out in the experimental setup. Referring to Fig. 1; LED 470 nm was replaced with LED 660 nm, LED 350 nm was turned on for excitation, 50:50 beamsplitter before the cameras was replaced with Dichroic Mirror Long Pass 650 nm and camera 1 was replaced with 340M-USB (Thorlabs, UK).

### Depth of focus

Depth of focus, DOF, is defined as the maximum z-height where the sample being imaged is accepted to be in focus. It’s given by the Berek formula^93^:

7 $$DOF\,[\mu m]=\frac{\lambda \,[\mu m]}{2{(NA)}^{2}}+\frac{\mathrm{Re}solving\,Power\,[\mu m]}{(NA)M}$$

where *λ* is the wavelength of the excitation light, NA is the numerical aperture of the lens and M is the total magnification of the system. *Resolving Power* can be subjective, however, it is usually given as 340 *μm*^94^ to estimate the DOF of the system. As can be seen from the Berek formula, higher NA decreases DOF; it’s a trade-off between resolution in the x-y plane versus the z-plane, therefore, DOF should be considered in experimental system design. In our study, the microchannel height was measured as 34 *μm*; the DOF for brightfield and fluorescent imaging were calculated as 75 *μm*, and 25 *μm*, respectively.

### Droplet detection with region of interest

Droplets can be optically detected using LabVIEW real time image acquisition and analysis tools. One such tool is called a region of interest (ROI). During real time acquisition of frames from a camera, ROIs are selected within the acquired frame to carry out further analysis. Built in function ‘IMAQ Quantify 2 VI’^95^ is used to extract the mean intensity, *I*_*m*_, of the current ROI. The lensing effect caused by the mismatch in the refractive indices of the fluid mediums gives rise to a dark ring forming around the droplet-oil interface. When this ring resides within the ROI, the mean intensity significantly drops below the automatically-set threshold, T. The intensity within the ROI is averaged over time, ${\overline{I}}_{m}$ to determine the threshold for droplet detection, $T=0.98{\overline{I}}_{m}$. This accounts for minor changes such as lighting, ROI, etc. to reliably detect droplets as they pass through the ROI. The multiplier could also be modified to account for ROI size and droplet frequency changes.

An important consideration, which justifies the usage of laser droplet detection system, is the discrepancy between camera frame rate and droplet velocity. When that happens, a droplet might appear before an ROI in one frame and after in the other which makes it a ghost droplet for the ROI detection system. This might be compensated by increasing the size of the ROI up to a certain limit. The laser detection system ensures a stable location for the droplet for image manipulation and actuation timing purposes.

### Laser spot size

Laser spot size is calculated by acquiring a non-saturated image of the laser itself without any other lighting. The intensity values from a line drawn along the axis of droplets are loaded into MATLAB’s Curve Fitting Tool and fitted with a Gaussian to calculate the full width at half maximum in pixels. This is later converted to microns using previously calibrated pixel size of 390 nm (40×).

## Data Availability

All connected data to this manuscript is available from the authors through reasonable request.
